# Decellularized human ovarian scaffold based on a sodium lauryl ester sulfate (SLES)-treated protocol, as a natural three-dimensional scaffold for construction of bioengineered ovaries

**DOI:** 10.1186/s13287-018-0971-5

**Published:** 2018-09-26

**Authors:** Ashraf Hassanpour, Tahereh Talaei-Khozani, Elias Kargar-Abarghouei, Vahid Razban, Zahra Vojdani

**Affiliations:** 1 Tissue Engineering Lab, Department of Anatomical Sciences, School of Medicine, Shiraz University of Medical Sciences, Shiraz, Iran; 2 Laboratory for Stem Cell Research, Department of Anatomical Sciences, School of Medicine, Shiraz University of Medical Sciences, Shiraz, Iran; 3 Molecular Medicine Department, School of Advanced Medical Sciences and Technology, Shiraz University of Medical Sciences, Shiraz, Iran; 4 Stem Cell Technology Research Center, Shiraz University of Medical Sciences, Shiraz, Iran; 5 Department of Anatomical Sciences, School of Medicine, Imam Hussain Square, Zand St, Shiraz, Fars 7134845794 Iran

**Keywords:** Artificial ovary, Decellularization, ECM-based scaffolds, Sodium lauryl ester sulfate, Tissue engineering

## Abstract

**Background:**

The increasing number of patients with ovarian insufficiency due to autoimmune disorders, genetic predisposition, or iatrogenic effects of treatment such as cancer therapies necessitates an urgent measure to find a safe and transplantable alternative ovary. A bioengineered ovary is one of the strategies on which the researchers have recently been working. An engineered ovary should be able to mimic the natural ovary aspects. Recent studies suggest that the decellularized organ-specific extracellular matrix-based scaffolds can serve as a native niche to bioengineering artificial organs. Therefore, we established a human decellularized ovarian scaffold based on a sodium lauryl ester sulfate (SLES)-treated process, as an optimized protocol.

**Methods:**

The human ovary samples were decellularized with 1% SLES for 48 h followed by DNase I in PBS for 24 h, and then thoroughly rinsed in PBS to remove the cell remnants and chemical reagents. Efficient cell removal was confirmed by DNA content analysis, hematoxylin and eosin, and Hoechst staining. Preservation assessment of the extracellular matrix structures was performed by immunohistochemistry, histological staining, and scanning electron microscopy. An MTT test was done to assess the in vitro scaffold’s cytocompatibility, and finally in vivo studies were performed to evaluate the biocompatibility, bioactivity, and secretion functions of the ovarian grafts made of primary ovarian cells (POCs) on the decellularized scaffolds.

**Results:**

Evidence provided by SEM, histochemical, and immunohistochemical analyses showed that the ovarian extracellular matrix was preserved after decellularization. Moreover, MTT test indicated the suitable cytocompatibility of the scaffolds. The in vivo assessment showed that the POCs kept their viability and bioactivity, and reconstructed the primordial or primary follicle-like structures within the scaffolds after transplantation. Immunostaining characterized somatic cells that were capable of expressing steroid hormone receptors; also, as a marker of granulosa cell, inhibin-α immunostaining demonstrated these cells within the grafts. Additionally, hormone assessment showed that serum estradiol and progesterone levels were significantly higher in ovariectomized rats with ovarian cells-seeded grafts than those with or without decellularized scaffold grafts.

**Conclusions:**

A human ovary-specific scaffold based on a SLES-decellularized protocol as a biomimicry of the natural ovarian niche can be an ideal scaffold used to reconstruct the ovary.

**Electronic supplementary material:**

The online version of this article (10.1186/s13287-018-0971-5) contains supplementary material, which is available to authorized users.

## Background

Ovary is an endocrine organ of the female reproductive system that has two important interrelated functions: gametogenesis and steroidogenesis [1]. Ovarian dysfunctions present multiple medical and psychosocial challenges for the affected women, like infertility problems, and menopause-like symptoms. Aside from reproductive function, ovarian hormones significantly influence other organs like the breast, vessels and bones [2, 3].

In recent years, the steadily increasing number of female cancer survivors who suffer from therapy-induced ovarian failure [4], as well as patients suffering ovarian insufficiency due to autoimmune disorders, genetic predisposition, or treatments for some diseases such as rheumatologic and chronic kidney diseases [5], reveal an urgent need for a safe and transplantable alternative ovary. Construction of bioengineered ovary is one of the strategies on which the researchers have been working recently.

Nowadays, there is growing interest in using decellularized extracellular matrix (ECM)-based scaffolds as a promising opportunity in the field of tissue engineering and regenerative medicine. In addition, decellularized ECM is a useful alternative in vitro model for studying the comprehensive roles of the ECM because it retains exclusively native biochemical characteristics and ultrastructural architecture [6]. The decellularized organ-specific ECM scaffolds can be followed by repopulation with suitable cells [7] or applied as bioinks for three-dimensional bioprinting applications [8–10] to create engineered organs. Another application of decellularized ECM is improvement in the biological properties of synthetic materials [11] or fabrication of hydrogels [12–15]. Decellularization techniques have been successfully used for a variety of organs including the heart [16], blood vessels [17], urethra [18], urinary bladder [19], kidney [20, 21], trachea [22], lung [23], skeletal muscle [24], liver [25], and gastrointestinal tract [26, 27].

The ovarian ECM plays an essential role not only as a framework that provides the architectural support, but also as a specialized microenvironment for regulating cellular activities and development, including cell proliferation and survival, steroidogenesis, regulation of cell aggregation and morphology. The ECM also acts as a reservoir for bioactive molecules like growth factors and cytokines, and influences the follicle formation, maturation, and fate [28–30]. Therefore, decellularized ovarian ECM as a native scaffold can provide a great three-dimensional microstructure for regenerative efforts to support such functions.

In 2015, the initial steps of decellularization of human and bovine ovary were performed, using sodium dodecyl sulfate (SDS) as an ionic detergent to remove the cellular content [31]. One concern with SDS- based protocols, especially those with high detergent concentrations or long-term exposures, is the disruption of native ECM compositions and architecture that has deleterious effects on the following scaffold recellularization [32–34].

Sodium lauryl ester sulfate (SLES) is a milder anionic detergent than SDS. Kawasaki et al. showed that SLES significantly reduced inflammation and thrombogenicity in the transplanted decellularized heart and kidney [35]. In addition, SLES showed better preservation of proteoglycans, cytokines (such as basic FGF) and ECM microstructures, including ECM laminar array and basement membranes around the vasculature, than SDS. These results indicated that SLES-treated decellularized scaffolds could be superior to those treated with SDS as the niche for stem cell differentiation [35, 36].

Inasmuch as the detergent used for whole-organ or tissue decellularization is an essential factor in preparation of a decellularized scaffold with structural ECM integrity, we investigated a novel SLES-based protocol for optimizing the decellularization of human ovaries. Then, we evaluated the in vivo biocompatibility and efficiency of ovarian implants made of primary ovarian cells on the decellularized scaffolds to restore the ovarian hormones and induce puberty in ovariectomized immature rats.

In this study, the human ovarian tissue was obtained from young female-to-male transgender people (trans men) undergoing sex reassignment surgery who were potentially excellent voluntary donors for experimental researches on human ovaries [37–39].

## Methods

### In vitro assessments

#### Human ovarian tissue collection

Human ovarian tissues were obtained from five 18–35 year old patients undergoing sexual reassignment surgery (hysterectomy and bilateral salpingo-oophorectomy) by laparoscopy after informed consent forms were taken. The entire ovaries were transported to the laboratory on dry ice. Upon arrival, the ovaries were rinsed in phosphate-buffered saline (PBS); then, the parts thermally damaged due to electrocoagulation were removed and the rest of the ovaries was frozen at −80 °C.

#### Decellularization process

For decellularization, the ovaries were bisected along the hilus and cut into manageable strips. Then, we separated the medulla from the cortex to prepare ~2.0 mm-thick tissue slices of the cortex. Some pieces were randomly collected for assessment as an intact tissue and the rest were decellularized with 1% SLES (Kimia Sanaat Ataman Co., Tehran, Iran) for 48 h at 18–20 °C on a magnetic stirrer at 100 RPM and subsequently 500 U/mL of deoxyribonuclease (DNase I) (Sigma-Aldrich, Gillingham, UK) in PBS for 24 h at 36 °C. Moreover, two additional ovaries were considered for whole-organ decellularization. The ovaries were bisected and then decellularized in 1% SLES for 30–40 days. Each step was followed by rinsing several times in PBS to remove the cell remnants and chemicals reagents. Decellularized and intact tissues were either fixed in 10% neutral-buffered formalin, 2.5% gluteraldehyde, or kept at −80 °C or in sterile PBS at 4 °C for subsequent analysis.

#### Histological assessments

Formalin-fixed decellularized ovarian scaffolds and intact tissues were embedded in paraffin and sectioned at 5–10 μm thickness. For confirmation of efficient cell removal, the sections were stained with hematoxylin and eosin (H&E), and Hoechst. The slides were examined by light (Olympus BX61, Tokyo, Japan) and fluorescent (Olympus, BX51) microscopes equipped with a digital camera (Olympus DP73), respectively.

ECM structures were qualitatively validated with the Heidenhain’s AZAN and Masson Trichrome staining for collagen fibers, Alcian blue (pH 2.5) for glycosaminoglycan (GAGs) and Gomori's aldehyde-fuchsin for elastic fibers.

#### DNA content analysis

DNA quantification assay was performed using a QIAamp^®^ DNA Blood and Tissue Mini Kit (Qiagen GmbH, Hilden, Germany) according to the manufacturer’s instructions, and the DNA yield (ng/μL) was quantified spectrophotometrically (the optical density (OD) at γ = 260 nm), using the NanoDrop® ND-1000 (Nanodrop Technologies Inc., Wilmington, DE, USA).

#### Immunohistochemistry

Preservation assessment of collagen types I and IV, fibronectin, and laminin was performed by immunohistochemistry. Briefly, fresh unfixed frozen sections were prepared at 7 μm thickness. Quenching of endogenous peroxidase activity was done by 3% H_2_O_2_ diluted in methanol for 20 min. Nonspecific binding sites were blocked with 300 μL PBS containing 10% goat serum and 5% bovine serum albumin (BSA) for 1 hour at room temperature and, then, the sections were incubated with biotinylated anti-collagen type I (1:250; ab6577), anti-collagen type IV (1:500; ab6581), anti-fibronectin (1:250; ab6584) and anti-laminin (1:100; ab6571) antibodies overnight at 4 °C (All from Abcam PLC, Cambridge, MA, USA). After washing, 200–400 μL streptavidin-HRP (1:10000; Abcam, USA; ab7403) was added to each section and incubated for 20–30 min at room temperature. Eventually, DAB + chromogen-substrate system (1:50; Dako, Glostrup, Denmark; K3467) and hematoxylin counterstaining were used for detection. A matched similar protocol was done except using primary antibodies incubation as a technical negative control.

#### Scanning electron microscopy (SEM)

For ultrastructural assessment, frozen decellularized samples were lyophilized overnight (Christ Alpha 2–4 LD-plus, Osterode am Harz, Germany). Sample preparation for intact ovaries and cell-containing scaffolds was based on a previously described method used by Kashi et al. [40]. Briefly, the samples were fixed using 2.5% glutaraldehyde (Sigma-Aldrich, St. Louis, MO, USA) plus 4% formaldehyde (Sigma-Aldrich, USA) in 0.1 M PBS (pH 7.4) at 4 °C overnight and then gradually dehydrated via an increasing graded series of ethanol. After that, the samples were immersed respectively into 1:2 and 2:1 Hexamethyldisilizane (HMDS; Merck, Kenilworth, NJ, USA): absolute ethanol for 20 min and then 100% HMDS solution overnight to air-dry in a fume hood. The samples were coated with a thin layer of gold, using Q150R- ES sputter coater (Quorum Technologies, London, UK) and imaged using an VEGA3 microscope (TESCAN, Brno, Czech Republic) at 10 kV accelerating voltage.

#### Cytotoxicity assessment

To assess in vitro decellularized ovarian scaffold’s cytocompatibility, human Wharton’s jelly mesenchymal stem cells (HWJMSCs) were seeded onto each scaffold and cultured at 5% CO_2_ and 37 °C. Prior to seeding, the scaffolds were cut into 5 mm diameter discs, lyophilized and then sterilized by UV light (wavelength: 253.7 nm) each side for 20 min; finally, we immersed them in the DMEM/F12 medium (Shell Max; A2930) containing 10% fetal bovine serum (FBS, Gibco, Paisley UK), 2 mM L- glutamine (Bioidea, Tehran, Iran), 100 U/mL penicillin and 100 μg/mL streptomycin (Gibco) for 2 h at 5% CO_2_ and 37 °C. At the third passage, HWJMSCs were seeded at a density of 1.0 × 10^6^ cells/scaffold. Cellular viability and proliferation were determined using 3-(4, 5-dimethylthiazolyl-2)-2,5-diphenyltetrazolium bromide (MTT) assay (M5655; Sigma-Aldrich) after 1, 3 and 7 days of culturing. The seeded scaffolds were removed and placed in a clean well on a 24-well plate; then, the MTT solution (1 mg/mL of MTT in DMEM) was added and the cultures were incubated for 3 h. Formazan crystals were dissolved by 300 μL dimethyl sulfoxide (Sigma-Aldrich) for 15 min. The optical density (OD) was measured at a wavelength of 595 nm. A conventional two-dimensional monolayer culture system on polystyrene 24-well dish was used at the same cell density as the control.

To visualize the seeded cells, the cells containing scaffolds were fixed in 2.5% glutaraldehyde and prepared for SEM preparation as described above after 21 days.

### In vivo assessments

#### Extracting the primary ovarian cells and seeding on the scaffold

According to Campbell et al.’s protocol [41] with some modifications, primary ovarian cells were isolated from 8-week female rats and seeded onto the scaffolds. Briefly, the ovaries were incubated in 6.8 mM EGTA + 0.2% BSA in the culture medium for 15 min at 37 °C followed by incubation in hypertonic sucrose solution (0.5 M sucrose with 1.8 mM EGTA and 0.2% BSA) for 5 min at 4 °C. Then, the ovaries were squeezed by a blunt spatula and punctured by a fine needle to release the primary ovarian cells into the culture medium and centrifuged at 1500 RPM for 5 min. The cell pellet was washed with fresh medium and the cells were resuspended with small pipettes in 200 μL of DMEM/F12 medium. Afterwards, the number of viable cells was checked by Trypan blue and the cells were cultured on fibronectin-coated plates in plating medium of DMEM/F12 (Shell Max; A2930) supplemented with 1 × insulin-transferrin-selenium (Sigma-Aldrich), 12.75 mM HEPES (pH 7.4; Sigma-Aldrich) 1× penicillin/streptomycin (Gibco), 10% FBS (Gibco) and 40 mg/mL hydrocortisone (Sigma-Aldrich) at 37 °C and 5% CO_2_ overnight. On the next day, after washing away the debris and any nonadherent follicles or oocytes with PBS, the presence of the ovarian stroma cells (granulosa and theca cells), adherent small follicles (primary and primordial follicles), denuded oocytes and cumulus oocyte complexes was confirmed by an inverted microscope. The ovarian cells were then harvested by 0.025% Trypsin/EDTA solution (T3924, Sigma-Aldrich), seeded onto the scaffolds by simple pipetting at a density of 2.0 × 10^6^ cells/scaffold and cultured with 400 μL of plating medium on 24-well plates at 37 °C and 5% CO_2_ for 1 day. Viability of ovarian cells was assessed by MTT test on some randomly selected seeded scaffolds before grafting.

#### Animal model

Four-week immature female Sprague-Dawley (40–55 g) rats were obtained from the central animal laboratory of Shiraz University of Medical Sciences and treated in accordance with the university ethics committee guidelines on the standard animal care.

Sixteen rats were divided randomly into ovariectomized (OVX) (*n* = 12) or sham-operated (non-OVX) (*n* = 4) groups. All animals were anesthetized with ketamine/xylazine (80/5 mg/kg body weight, intramuscular) and bilateral ovariectomy was performed. After a 14-day recovery period, the vaginal orifice of the rats was checked as a criterion of successful ovariectomy. The ovariectomized rats were then randomly assigned into three groups including ovariectomized control (OVX-C) without further treatment, those receiving decellularized scaffolds seeded by primary ovarian cells (POCs-DS), and those receiving decellularized scaffolds (DS). Eight ovariectomized rats were reopened and the grafts were sutured onto the renal fat pad bilaterally. Four weeks after the surgery, vaginal patency was rechecked; then, under deep anesthesia with chloroform, the blood samples were collected by cardiac puncture. Finally, the grafts were removed and fixed in 4% paraformaldehyde for further analyses.

#### Serum hormone assay

Serum levels of 17β-estradiol (E_2_) and progesterone (P_4_) were measured using commercially available enzyme immunoassay kits (ELISA, #RE52041, range of 9.7–2000 pg/mL; #RE52231, range of 0–40 ng/mL respectively; IBL, Hamburg, Germany), following the manufacturers’ instructions. The analytical sensitivity for the E_2_ assay was 10.60 pg/mL; it is reported to have 6.86% cross-reactivity with estrone, 2.27% with estriol, and less than 0.05% with other steroid hormones compared to 100% for E_2_. The analytical sensitivity for the P_4_ assay was 0.045 ng/mL and it is reported to have less than 1.2% cross-reactivity with other steroid hormones. Microtiter wells are coated with a polyclonal rabbit anti-E_2_ (-P_4_) antibody. Endogenous E_2_ (P_4_) of a sample competes with an E_2_ (P_4_)-horseradish peroxidase conjugate for binding to the coated antibody. The intra- and inter- assay coefficients of variation (CVs) for the E_2_ assay were 6.9% and 9.2%, respectively; for the P_4_ assay they were 5.8% and 4.7%, respectively. Assays were performed at Professor M. Saeb Specialized Hormone Laboratory, Shiraz, Iran. The samples were assayed in duplicate, and all the subjects’ samples were assayed together. The absorbance at each assay was read at a wavelength of 450 nm with a plate reader (Stat Fax 2100, Awareness, Phoenix, AZ, USA).

#### Histological and immunohistochemical assessments of transplanted grafts

H&E staining was performed to evaluate the basic histomorphology and immunohistochemistry was done to detect the specific markers of the ovarian cells on the transplanted grafts. Immunostaining was performed by the avidin-biotin-peroxidase complex method. Briefly, deparaffinized and rehydrated sections were incubated with 3% H_2_O_2_ diluted in methanol to quench endogenous peroxidase, and antigens retrieval was done with citrate buffer at a pH of 6 on a heater for 30 min. After blocking of nonspecific binding sites, the sections were incubated with primary monoclonal antibodies including anti-estrogen receptor (ER), anti-progesterone receptor (PR) (prediluted, both master diagnosticá; MAD-000306QD-7, MAD-000670QD-7 respectively) and anti-inhibin-α (ready-to-use; Dako; IR058) for 1 h followed by incubation with post-primary block and polymer for 30 min and visualized with DAB (Dako; K3467). The samples were counterstained with hematoxylin.

In addition, an anti-deleted in azoospermia-like (DAZl) antibody (1:100; Abcam; ab34139) was used and visualized with an Alexa-Fluor^®^488-conjugated secondary antibody (1:500; Abcam; ab150077) to detect the possible presence of germ cell-like cells (GCLCs) in the transplanted DS sections.

The number of follicles in the POCs-DS grafts was counted in the five sections per graft using ImageJ (http://rsb.info.nih.gov/ij/). First, five pictures of each section were randomly taken by an optical microscope (Nikon E200, Tokyo, Japan) equipped with a ×20 objective with an Samsung digital camera (SCB-2000), and then the images were imported into ImageJ software, and the number of follicles was counted in each image. Then, the number of follicles and total analyzed areas of each graft were measured. Follicles that exhibited an organized granulosa cell layer around a visible, round and generally centralized oocyte were considered as healthy ones.

### Statistical analysis

Quantitative data were expressed as the mean value ± standard error of mean (S.E.M). Independent samples *t* test for DNA quantification and one-way analysis of variance (ANOVA) followed by Tukey’s multiple comparison test for hormone and MTT assays data were performed. The data were analyzed using SPSS software 17.0 (SPSS Inc., Chicago, IL, USA). A *p* values of < 0.05 was considered as significant.

## Results

### In vitro assessments

#### Decellularized scaffold assessments

**Fig. 1:**
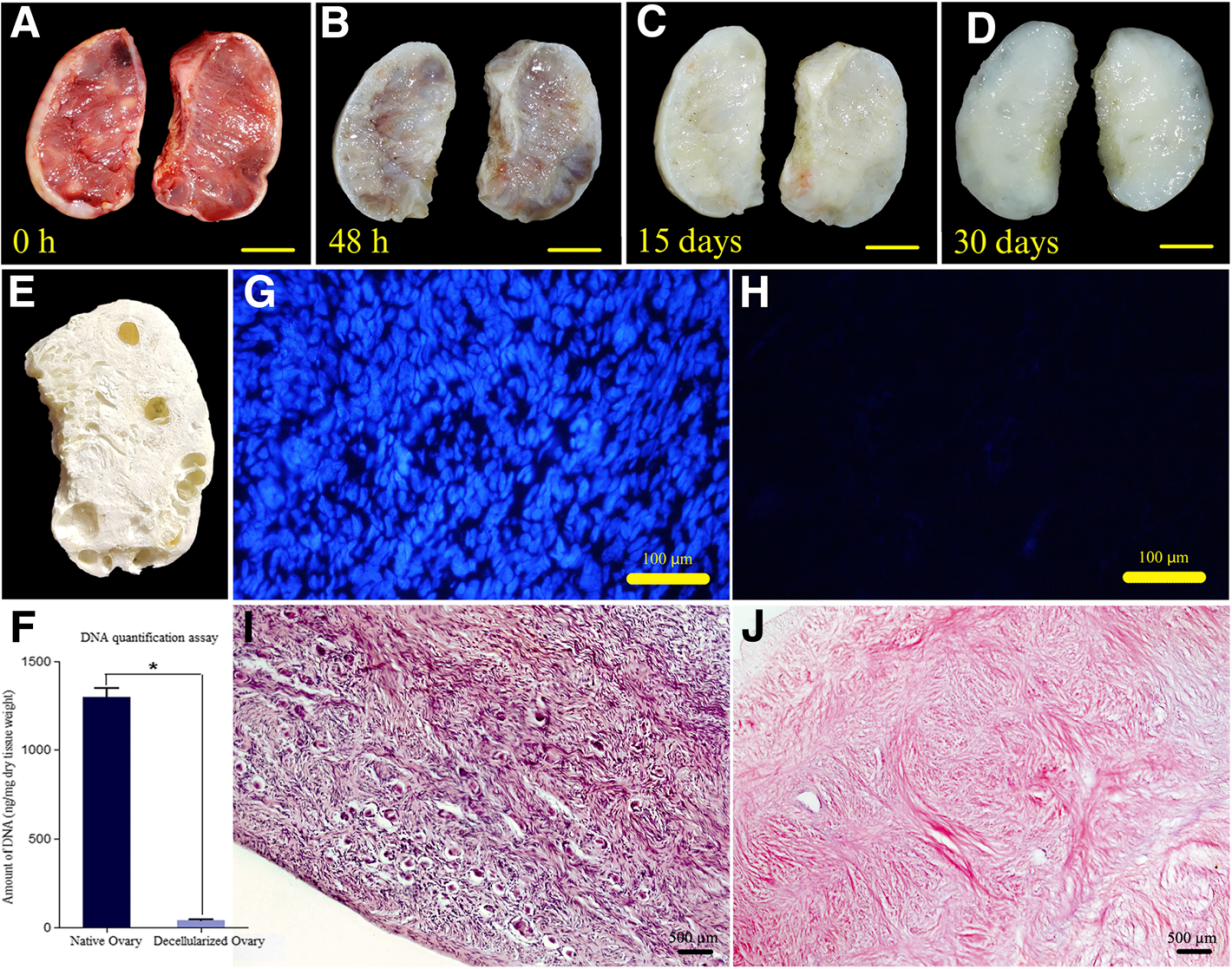
Chronological macroscopic and microscopic changes of the human ovary during SLES-based decellularization process. **a-d** The color of the bisected ovarian samples turned from red to white while the samples preserved their shape and homogeneity. **e** A lyophilized decellularized ovarian scaffold with visible pores once populated by growing follicles; scale bars: 10 mm. **g-j** Hematoxylin and eosin (**i** and **j**), and Hoechst (**g** and **h**) staining of intact (**g** and **i**) and decellularized (**h** and **j**) ovary showed it was devoid of nucleic materials. **f** A drastic decrease in DNA content after decellularization. (Data are expressed as the mean ± standard error of the mean (SEM), *N* = 3 per group, *Indicates significant difference, *p* = 0.0001)

During decellularization process, the color of the human ovaries turned from red to white and became semi-transparent. The samples preserved their shape and homogeneity without any deformation (Fig. 1a-e, respectively).

Both H&E and Hoechst staining showed that the scaffolds appeared to be devoid of nucleic materials (Fig. 1g-j). In addition, DNA quantification assay showed a drastic decrease in the DNA content after decellularization (40 ± 7.33 ng/mg dry tissue weight in decellularized samples compared to 1296.26 ± 52.59 ng/mg dry tissue weight in intact samples (*N* = 3 per group, *p* = 0.000), (Fig. 1f).

Histochemical assessments revealed preservation of the ECM content. Both Heidenhain’s AZAN and Masson Trichrome staining demonstrated the persistence of collagen fibers. Immunohistochemistry also confirmed the collagen I preservation. Collagen I, as the most abundant protein of the ovarian interstitial matrix, was present throughout the decellularized cortex and medullary regions with higher concentrations in the tunica albuginea and follicular compartments.

**Fig. 2:**
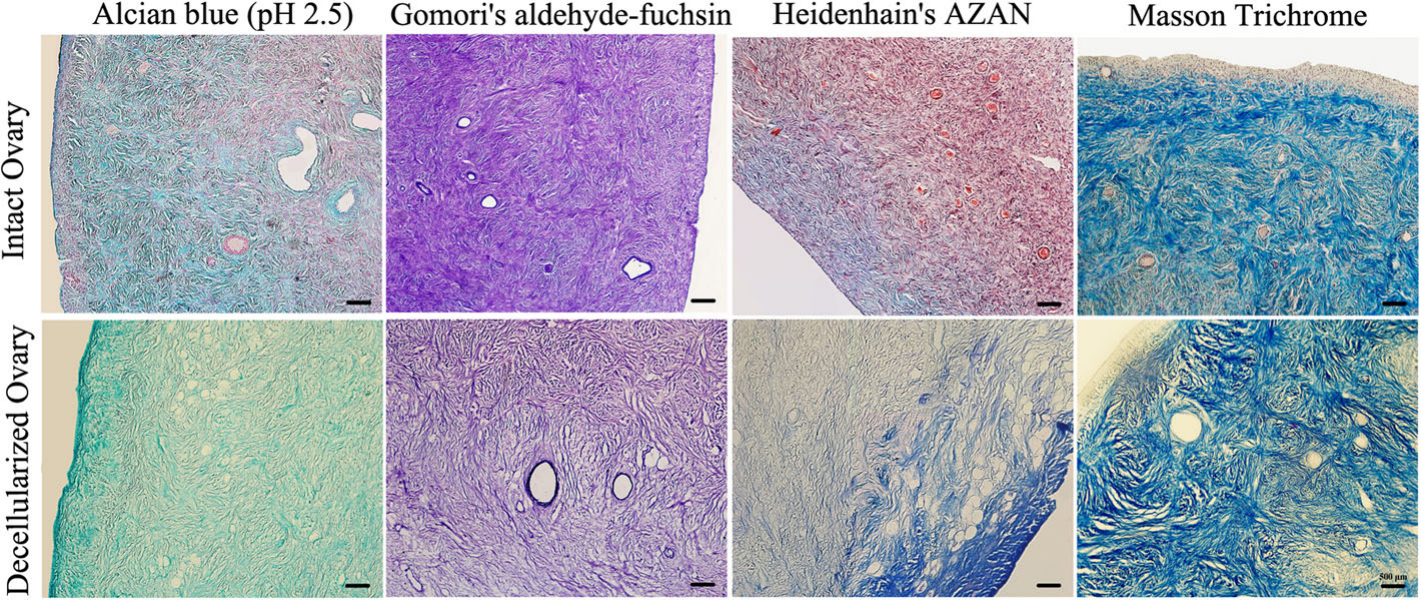
Extracellular matrix structure and composition preservation. Alcian blue (pH 2.5) and Gomori's aldehyde-fuchsin for detection of the glaycosaminoglycans and elastic fibers, respectively, and Heidenhain’s AZAN and Masson Trichrome staining for the detection of collagen fibers. Scale bars: 500 μm

Gomori's aldehyde-fuchsin and Alcian blue (pH 2.5), respectively, revealed that the elastic fibers and GAGs were kept after the decellularization process (Fig. 2). The GAGs concentration was more in the ovarian cortex. Elastic fibers scattered throughout the ovary, but they presented especially in the media of the vessels.

**Fig. 3:**
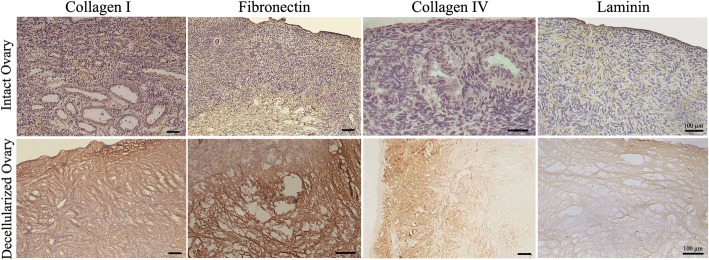
Immunohistochemical validation of extracellular matrix proteins that have been preserved in the decellularized matrix. Collagen I, fibronectin, collagen IV, and laminin were appropriately detected in the decellularized matrix with similar distribution pattern as in intact tissue. Scale bars: 100 μm

The appropriate preservation of collagen IV, fibronectin, and laminin in decellularized scaffold was also shown by immunohistochemistry staining. Laminin and collagen IV were observed throughout the decellularized cortex. Collagen IV was plentiful in the theca cells compartment and low-level in the medullary stroma. Laminin was localized in the basal lamina of the ovarian surface epithelium and the surrounding part of the follicular granulosa cells and blood vessels. Fibronectin was observed in the tunica albuginea, theca cells compartment and the medullary stroma. Interestingly, the distribution pattern of these proteins in the decellularized ovary matrices was similar to that of intact tissues (Fig. 3).

#### SEM assessment

**Fig. 4:**
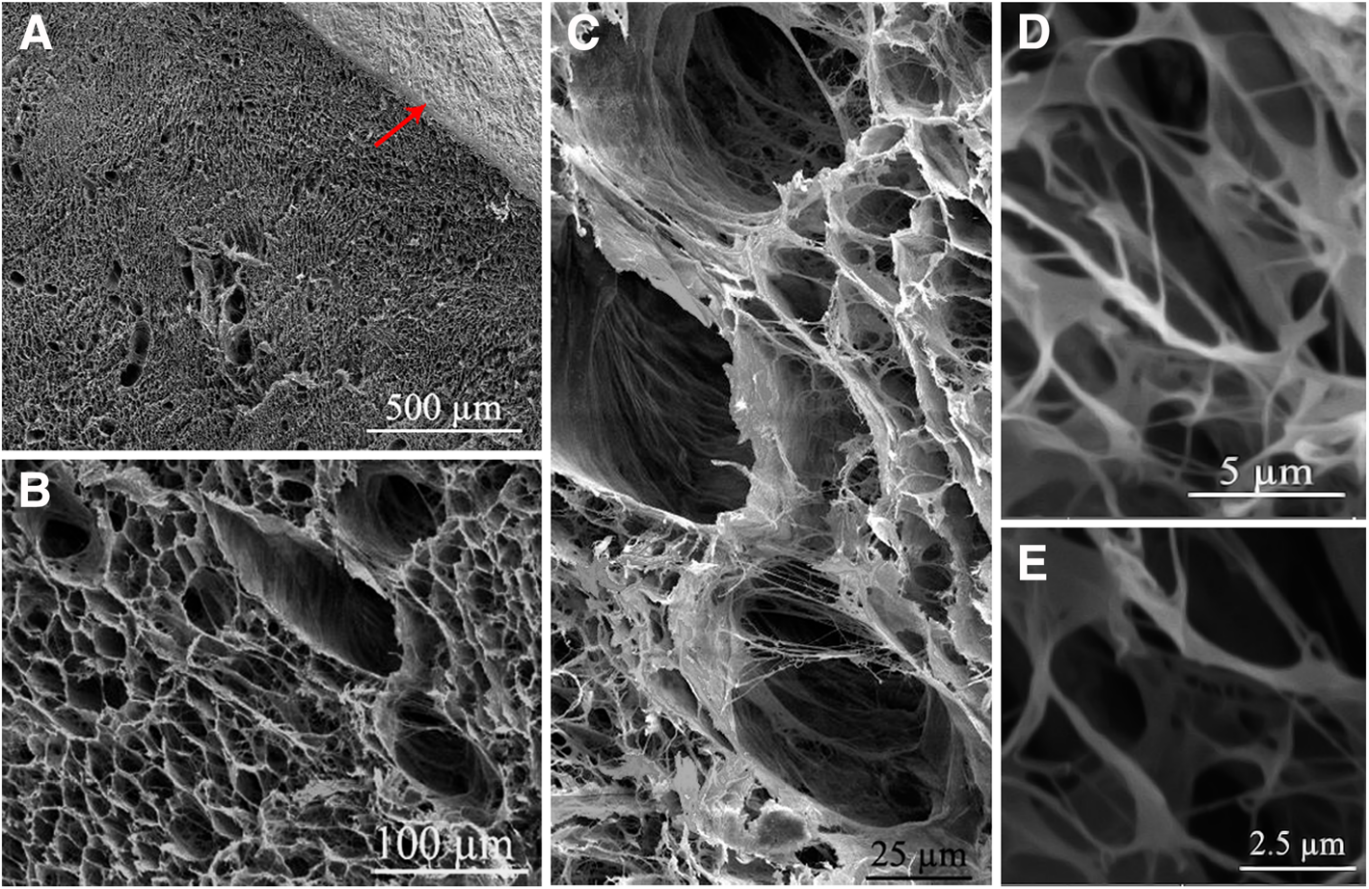
Scanning electron microphotographs of decellularized ovarian scaffolds. **a-e** The efficient removal of cells and good preservation of three-dimensional structures and integrity are revealed after decellularization. **a** The ovarian surface epithelium is visible on the ovarian cortex (*red arrow*). **c-e** A complex fiber network with porous structures once populated by different cell types is visible, and the collagen fibers orientation and structure are almost unaffected

Along with light microscopy findings, SEM assessment showed microarchitecture integrity and efficiency devoid of cells after decellularization. Lower magnification microphotographs of decellularized ovarian scaffolds showed a complex fiber network with porous structures once populated by different cell types. In higher magnifications, the ECM framework appeared intact in different regions such as tunica albuginea and those once occupied by primordial and growing follicles as well as vessels. SEM also revealed that the collagen fibers orientation and structures were almost unaffected (Fig. 4).

#### MTT assay

**Fig. 5:**
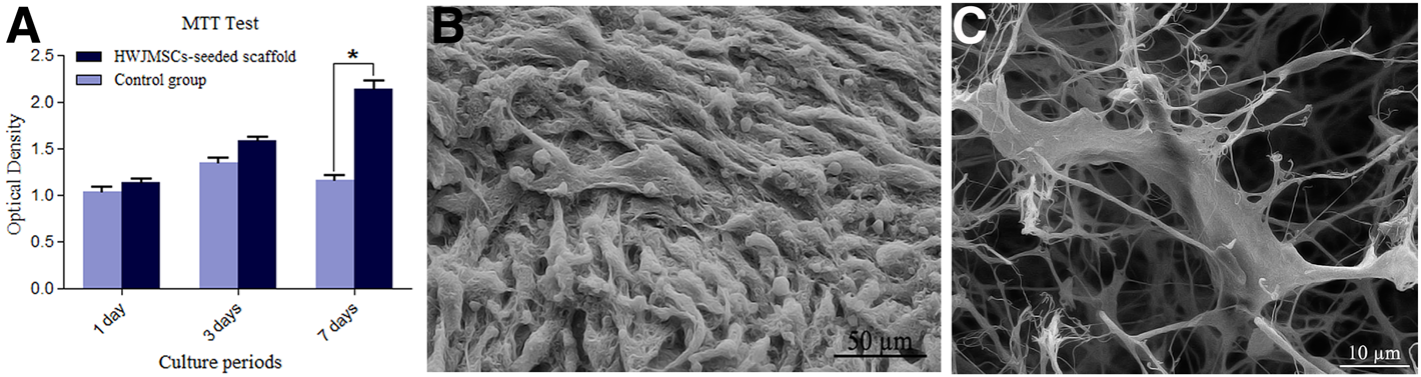
Cytocompatibility assessment of decellularized ovarian scaffold. To assess soluble toxicity, HWJMSCs at a density of 1.0 × 10^6^, we seeded the cells onto each scaffold and cultured 1, 3 and 7 days. **a** MTT test showed that the cell proliferation on the decellularized scaffold was parallel with the control culture up to day 3, whereas, at longer culture intervals, a significant higher optical density value was detected in the seeded decellularized scaffolds than in the control group. Data are expressed as the mean ± standard error of the mean (SEM), *Indicates significant difference, *p* = 0.000). SEM micrographs revealed that the cells were attached on the surface of the scaffolds (**b**), and also they penetrated deep into the matrix (**c**). *HWJMSCs* human Wharton’s jelly mesenchymal stem cells

To determine the cytocompatibility of decellularized scaffold, we assessed the viability and proliferation of HWJMSCs. MTT test showed that the seeded HWJMSCs on the decellularized scaffolds were viable and comparison of the OD values of eluted formazan in various dates of culture revealed that the cells could proliferate properly in the scaffold. At the initial phase of culturing, the cell proliferation on the decellularized scaffold was parallel with the control culture, whereas, at longer culture intervals, a significant higher OD values were detected in the scaffolds, indicating the higher proliferation rate of the HWJMSCs in the decellularized scaffold than in the two-dimensional conventional culture system (Fig. 5a).

Furthermore, SEM micrographs revealed that the cells not only attached on the surface of the scaffolds (Fig. 5b), but also penetrated deep into the matrix (Fig. 5c). Thus, cytocompatibility assessment showed that the decellularized ovarian scaffolds were cell compatible.

### In vivo assessment

In vivo studies were performed to evaluate the biocompatibility, bioactivity, and secretion functions of the ovarian cells-seeded scaffold after transplantation. Our findings showed fairly good adaptation of the grafts at recipients. None of the rats died and no major complication was observed as the result of the procedure during 4 weeks of follow-up after the surgery.

#### Histological and immunohistochemical assessment of the transplanted grafts

**Fig. 6:**
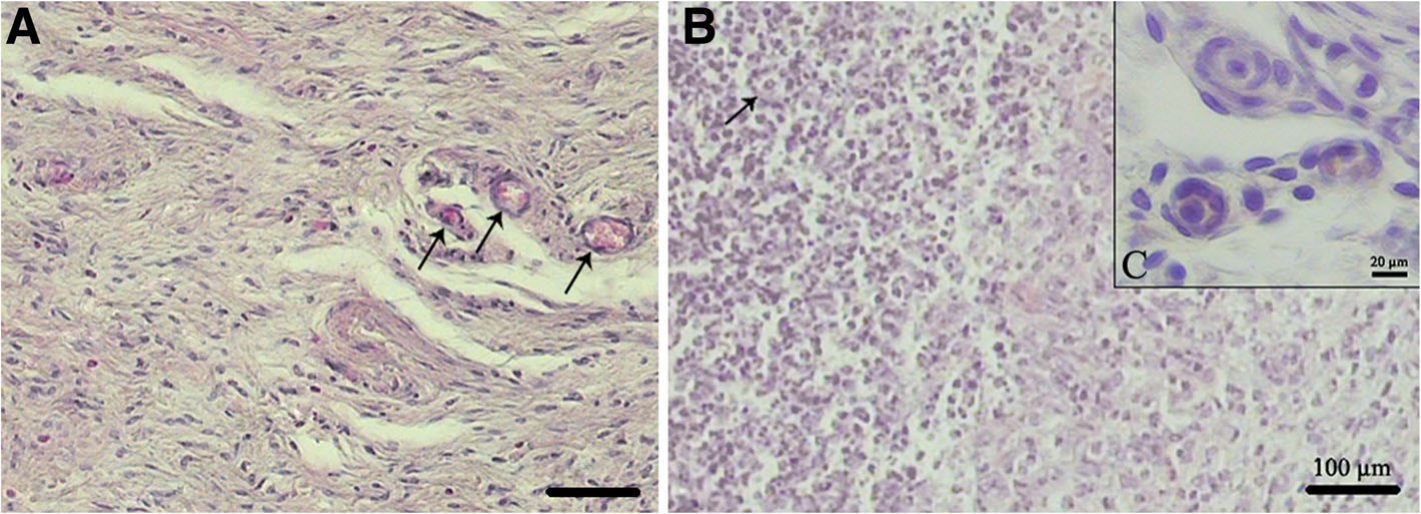
Histological assessments of DS (**a**) and POCs-DS (**b**) transplanted grafts. **a** H&E staining revealed the presence of host cells and suitable neovascularization (*black arrows*). Scale bar: 100 μm. **b** A high number of viable ovarian cells population in the POCs-seeded scaffolds. POCs were found in clusters inside the scaffolds and several primordial or primary follicle-like structures were identified *(black arrow* and **c**). Scale bar: 100 μm. **c** Lightly stained round oocytes were surrounded by different numbers of granulosa cells in close contact with each other. Scale bar: 20 μm. *DS* decellularized scaffolds, *POCs* primary ovarian cells, *POCs-DS* primary ovarian cells-seeded decellularized scaffolds

In macroscopic observations, there was no sign of graft rejection (Additional file 1). However, histological assessment by H&E staining revealed the presence of host cells such as neutrophils, lymphocytes, macrophages, fibroblasts, and endothelial cells in both groups receiving POCs-DS and DS (Fig. 6). Also, as indicated by the presence of red blood cells, neovascularization was seen in the grafts (Fig. 6a; Additional file 1). Furthermore, histological sections showed a high number of viable ovarian cells population in the POCs-seeded scaffolds. POCs were found in the clusters inside the scaffolds, and several primordial or primary follicle-like structures were identified. The structures showed lightly stained round oocytes surrounded by different numbers of granulosa cells in close contact with each other (Fig. 6b). Analysis using ImageJ showed that in a mean area (997,501.38 μm^2^) of the grafts, the primordial and primary follicles were 56.48% and 43.52% of the follicle population, respectively. Of the eight grafts, seven showed the presence of follicles, but one graft contained only ovarian stromal cells, and we observed no secondary or antral follicles in the selected grafts’ sections (Table 1).

**Table 1:** The number of follicles in the POCs-DS grafts 4 weeks post-transplantation

Rats	Grafts	Follicles				
		Primordial follicles	Primary follicles	Total surface area analyzed (μm^2^)	Number of primordial follicles/μm^2^	Number of primary follicles/μm^2^
1	Right	5	7	1,012,500	4.9 × 10^−6^	6.9 × 10^− 6^
	Left	7	9	984,236	7.1 × 10^−6^	9.1 × 10^− 6^
2	Right	11	4	1,142,153	9.6 × 10^−6^	3.5 × 10^− 6^
	Left	7	10	1,000,971	7 × 10^−6^	1 × 10^− 5^
3	Right	14	6	924,048	1.5 × 10^−5^	6.5 × 10^−6^
	Left	9	6	1,011,689	8.9 × 10^−6^	5.9 × 10^− 6^
4	Right	0	0	1,004,704	0	0
	Left	8	5	899,710	8.9 × 10^−6^	5.6 × 10^−6^
Mean ± SD		7.63 ± 4.14	5.88 ± 3.09	997,501.38 ± 72,258.24	7.7 × 10^− 6^ ± 4 × 10^− 6^	5.9 × 10^− 6^ ± 3 × 10^− 6^

*SD.* standard deviation

**Fig. 7:**
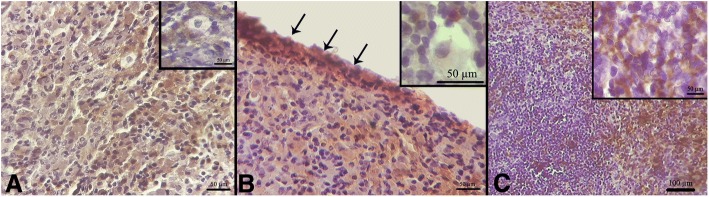
Immunohistochemical assessments of POCs-DS (A-C) transplanted grafts. **a** Inhibin-α immunostaining for the detection of the granulosa cells. These cells (in *brown*) were scattered throughout the graft and some of them were organized into a follicle-like pattern (inset, **a**); Scale bars: 50 μm. **b** ER-positive brown cells were observed within the POCs-DS grafts. Strong staining intensity was observed in the cells located peripherally on the scaffolds (*black arrows*) compared with those located more centrally (*inset*, **b**); scale bars: 50 μm. **c** PR-positive cells were observed within the POCs-DS grafts as well; scale bar: 100 μm. (*inset*, **c**): PR-positive cells around an oocyte; scale bars: 50 μm. *ER* estrogen receptor, *POCs-DS* primary ovarian cells-seeded decellularized scaffolds, *PR* progesterone receptor

Additionally, inhibin-α immunostaining as a marker of granulosa cell demonstrated these cells within the transplanted scaffold. They were scattered throughout the graft and also organized into a follicle-like pattern, as well (Fig. 7a).

**Fig. 8:**
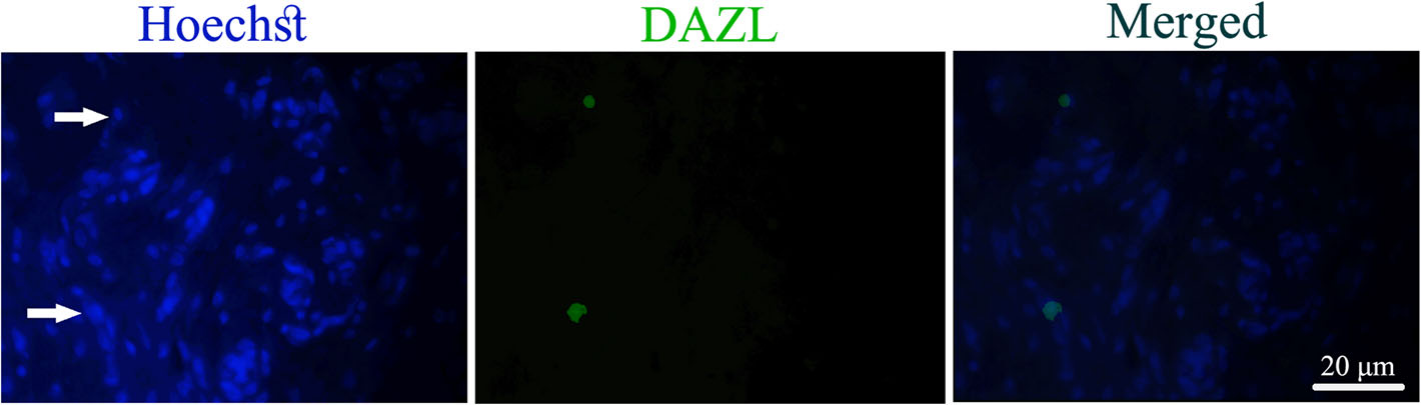
Immunofluorescent staining validation of the presence of DAZL-positive cells in DS grafts. *DAZl* anti-deleted in azoospermia-like, *DS* decellularized scaffolds

Also, PR- and ER-positive cells were observed within the POCs-DS grafts that indicates the expression of steroid hormone receptors in these cells (Fig. 7b, c). Interestingly, strong staining intensity was observed in the cells located peripherally on the scaffolds compared with those located more centrally (Fig. 7b). Finally, very few DAZL-positive cells were identified in the DS grafts that indicate presence of GCLCs (Fig. 8).

#### Serum hormone assays

Hormone assessment showed that serum estradiol and progesterone levels were significantly more in the POCs-DS and proestrus phase non-OVX groups than in the OVX-C and DS groups. In the POCs-DS group, transplantation led to progesterone restore up to physiological level; although estradiol was partly elevated, it was of no significance and was still below the physiological levels compared with that in the non-OVX group. In the animals receiving DS, estradiol and progesterone levels were statistically similar to those of the OVX rats (Table 2).

**Table 2:** Primary ovarian cells seeded on decellularized ovary scaffold restore serum estradiol and progesterone levels in ovariectomized rats after transplantation

	non-OVX	POCs-DS	OVX-C	DS
Estradiol (pg/mL)	66.47 ± 7.02^*^	41.69 ± 2.44**	19.54 ± 1.57	21.092 ± 1.69
Progesterone (ng/mL)	36.67 ± 1.79	31.17 ± 3.35^#^	7.51 ± 1.26	7.91 ± 2.67

Results are presented as mean ± S.E.M (standard error of the mean); n = 4 in each group. *Non-OVX* non-ovariectomized group, *OVX-C* ovariectomized control group, *POCs-DS* primary ovarian cells seeded decellularized scaffolds recipient group, *DS* decellularized scaffolds recipient group. Significance was calculated using one-way analysis of variance (ANOVA) with Tukey’s multiple comparison test. *p* values < 0.05 were considered as statistically significant

^*^Indicates *p* < 0.005, Non-OVX vs. the other groups

^**^Indicates *p* < 0.018, POCs-DS vs. OVX-C and DS

^#^Indicates *p* < 0.0003, POCs-DS vs. OVX-C and DS

Vaginal patency observations corroborated these findings. The vaginal orifice of OVX-C and DS animals was imperforate with a visible hymen, while the orifice of the rats receiving primary ovarian cells-seeded scaffolds was found to be open but less than that of non-OVX animals (Additional file 2).

## Discussion

To take the first steps toward fabricating an artificial ovary with the capability to effectively mimic the natural ovary environment and support ovarian stromal cells and oocytes, we established a human decellularized ovarian scaffold based on an SLES-treated protocol and showed that recellularized ovarian scaffolds were biocompatible and functional in vivo.

The ECM combinations such as type I and IV collagen, fibronectin, laminin, and GAGs are important in folliculogenesis. They play essential roles in regulating the oocyte maturation, follicle development and fate, steroidogenesis, and ovarian cells morphology [30]. An ideal decellularized tissue possesses exclusively native biochemical and biomechanical characteristics. Therefore, providing a standard protocol to preserve the constitutions of the cell-free ovarian ECM was one of main goals of the current study. Although SDS is routinely used in many decellularization protocols [32], a long-term treatment significantly alters the ECM, has a strong damaging effect on the ultrastructure of the native tissue, including reduction of the glycans and cytokines, is cytotoxic, has poor attachment property, and induces inflammation and thrombus formation after transplantation [32–34]. Previously, studies showed that SLES, a milder anionic detergent, could decrease the drawbacks related to SDS and may be a more suitable reagent for decellularization [35, 36]. Since the ovary does not have an extensive vasculature, immersion and agitation is an approach for decellularization, and depends on the size and thickness of the samples, immersion duration and the intensity of agitation will be different [42]. In this research, the decellularization process by SLES was preceded by a variety of agents, and durations were tested (data not shown); it was eventually found that immersing the samples in SLES for 48 h followed by DNase I in PBS for 24 h and finally excessive rinsing in PBS to remove the cell remnants was the optimal protocol.

Our data confirmed that this protocol is able to meet previously established decellularization criteria such as invisible nuclear material in the tissue sections and < 50 ng dsDNA/mg dry ECM weight [32]. Moreover, immunohistochemistry and histological staining showed that the ECM structure and composition were satisfactorily preserved after decellularization. The SEM also confirmed the efficient removal of the cells and good preservation of three-dimensional structures and integrity after decellularization.

Another important concern is the cytocompatibility of decellularized scaffolds. Residual cellular material and cytotoxic detergents within ECM may impair the subsequent recellularization and biocompatibility of an acellular scaffold both in vitro and in vivo [43].

HWJMSCs were viable and proliferated on the decellularized scaffold in parallel with the control group up to day 3, whereas, at longer culture intervals, a higher proliferation rate was detected in those growing in the scaffolds. The full cell confluence in the conventional two-dimensional culture system was believed to have occasioned lower proliferation rate at longer culture intervals. Furthermore, in the SEM analysis, not only the plentiful cells attached on the surface, but also some of them penetrated into the matrix. These all revealed that there were no indications that our decellularized scaffold displayed toxicity.

The ultimate goal of our research was to generate transplantable scaffolds that imitate the ovary microenvironment, providing an appropriate niche for POCs to be alive and functional in vivo. An engineered ovary should be able to effectively mimic the natural ovary aspects such as induction of the steroidogenesis and folliculogenesis; for this, it is necessary to utilize an appropriate scaffold that not only encloses and protects the follicles and ovarian cells, but also maintains bioactive factors that are essential for normal follicle development and oocyte maturation. Apart from that, such a scaffold should be degradable in order to allow neovascularization, exponential growth of the follicles, and cell migration and proliferation [44–46]. This study indicated that SLES-treated decellularized scaffold with specific properties (e.g. maximum resemblance to the in vivo conditions, suitable vessels formation and high compatibility) can be widely applied to construction of bioengineered ovary.

Several aspects of the oocyte growth and meiotic maturation are coordinated with granulosa cells-oocytes interactions. On the other hand, close interactions between the oocyte and the surrounding somatic cells are required for the follicle formation; proliferation and differentiation of granulosa cells; and steroid hormone production. Thus, an impaired oocyte-granulosa cell complex would lead to defective folliculogenesis and steroidogenesis [47–50]. Also, the ovarian ECM has various impacts on the oocyte maturation and granulose cell secretion [29]. For instance, GAGs cause the cumulus cells to produce cytokines, such as GFD9 [51] and also play key roles in in vitro cumulus cell expansion and oocyte maturation [52]. Besides, collagen hydrogel density and elasticity have been revealed to impact the folliculogenesis [53]. Thus, the natural amount of collagen with the naive elasticity in decellularized scaffolds may protect the folliculogenesis.

Herein, transplanted POCs kept their viability and reconstructed the primordial or primary follicle-like structures within the scaffolds. Immunostaining characterized the somatic cells that were active and capable of expressing steroid hormone receptors. The findings indicated that the scaffolds provided an appropriate niche for POCs to be alive and functional. Furthermore, hormone assays confirmed these results since ovariectomized rats with or without DS grafts had basal serum estradiol and progesterone levels, and the hormone levels in the rats receiving POCs-DS grafts markedly increased. The levels of these hormones were enough to induce vaginal patency as a criterion of pubertal transition in ovariectomized rats.

Histological investigation also showed functional blood vessels and capillaries that indicated suitable neovascularization in the grafts. Although some immune cells infiltrated inside the grafts, it is important to note that we used allogeneic cell transplantation into immunocompetent recipients, so minimal reaction is acceptable. Nonetheless, the inflammatory reaction was not enough to cause a gross rejection of the grafts.

We also found a few DAZL-positive cells in the DS grafts. DAZL is a member of the DAZ family proteins and is immunolocalized in the nucleus and/or in the cytoplasm of the germ cells. This family is expressed by the cells in most of gametogenesis stages and plays a key role in primordial germ cells (PGCs) development, differentiation, and maturation [54]. DAZL-positive cells were found in the adult female mouse bone marrow and have been suggested as an extra-gonadal source of germ cells. Depleted ovary with chemotherapy has been reported to rescue by bone marrow transplantation [55]. Besides, very small embryonic-like stem cells (VSELs) are postulated to be either involved in migration or as a possible origin of the DAZL-positive cells in DS grafts. VSELs are a primitive and pluripotent population of stem cells which are equivalent to PGCs. These cells migrate to all developing organs during early development and provide a backup population for tissue-committed stem cells in different adult organs such as the bone marrow, brain and pancreas. VSEL-positive cells are able to differentiate into embryonic cell lineages including oocyte-like structures [56–58]. Various studies have demonstrated that hematopoietic progenitor stem cells (HSCs) and VSELs can be mobilized into circulation, give rise to progenitors under stress or disease situations, contribute to organ repair, and maintain homeostasis [58–60]. Besides, Zbucka-Kretowska and colleagues revealed the effective mobilization of HSCs and VSELs by follicle-stimulating hormone (FSH) therapy in human [61]. In addition, a high-level of serum FSH following ovariectomy is proved by several studies [62–65].

Thus, in accordance with these findings, we postulated that migration of the cells expressing germ-line markers from bone morrow to DS grafts and their differentiation into GCLCs are possible and logical. However, more research is necessary to confirm these results.

## Conclusions

Herein, we showed that a human ovary-specific ECM scaffolds based on an SLES-decellularized protocol because of its specific properties (e.g., preservation of ovarian ECM structure and composition, in vivo and in vitro biocompatibility and neovascularization) is a promising candidate for reconstruction of bioengineered ovary as a biomimicry of the natural ovarian niche to support the ovarian stromal cells and oocytes.

### Future research directions

The future perspective of this study is fabricating an artificial ovary for restoring human fertility and ovarian function. We represent here the first steps toward this ultimate goal by establishing an effective decellularization protocol for human ovaries; it was shown that ovarian cells kept their viability and activity in this natural three-dimensional scaffold after 4 weeks of grafting. However, despite these promising results, it is important to emphasize that in vivo multiple time points and longitudinal in-depth studies are required to thoroughly analyze reproducibility and capability of the ovarian bioengineered grafts for restoration of fertility and endocrine functions.

Additionally, although in vivo serum estradiol and progesterone levels and immunostaining results were encouraging, long-term follow-up of estradiol, progesterone, inhibin, follicle-stimulating and luteinizing hormones as the key regulators of sex development and reproduction and also measurement of anti-Mullerian hormone as a valid and valuable marker of the number of small growing follicles and ovarian follicle reserves are required to assess the longevity and endocrine activity of the ovarian cell-seeded grafts.

In the current study, we recellularized only decellularized ovarian pieces, using rat ovarian follicles and cells; hence, significant hurdles to the recellularization of the whole organ scaffolds remain. In future research, the ovarian ECM scaffold can be repopulated with ovarian cells isolated from fresh or cryopreserved ovarian tissue or cells derived from oogonial stem cells (OSC), iPSC, or VSELs. Therefore, recellularization methods, culture conditions and preservation technologies also need to be improved.

## Additional files

Additional file 1: Macroscopic observation of the transplanted grafts. (A): The grafts (*yellow arrow*) were sutured onto renal (*green arrow*) fat pad of ovariectomized immature rats bilaterally. (B): Gross observation showed a vascular structure (*yellow arrow*) and there was no sign of rejection 4 weeks after the surgery. (TIF 1470 kb)

Additional file 2: Vaginal patency as a criterion of pubertal transition. The vaginal orifice of OVX-C animals was imperforated with a visible hymen (C), while the orifice of the rats receiving POCs-DS grafts (A) was found to be open, but less than that of the non-OVX animals (B). *OVX-C* ovariectomized control, *non-OVX* non-ovariectomized, *POCs-DS* primary ovarian cells seeded decellularized scaffolds. (TIF 6975 kb)

## Abbreviations

BSA: Bovine serum albumin
DAZl: Deleted in azoospermia-like
DNase: Deoxyribonuclease
DS: Decellularized scaffolds
ECM: Extracellular matrix
ER: Estrogen receptor
FBS: Fetal bovine serum
GAGs: Glycosaminoglycans
GCLCs: Germ cell-like cells
H&E: Hematoxylin and eosin
HSCs: Hematopoietic progenitor stem cells
HWJMSCs: Human Wharton’s jelly mesenchymal stem cells
MTT: 3-(4, 5-dimethylthiazolyl-2)-2,5-diphenyltetrazolium bromide
OD: Optical density
OVX: Ovariectomy
PBS: Phosphate-buffered saline
PGCs: Primordial germ cells
POCs: Primary ovarian cells
PR: Progesterone receptor
SDS: Sodium dodecyl sulfate
SEM: Scanning electron microscopy
SLES: Sodium lauryl ester sulfate
VSELs: Very small embryonic-like stem cells

## References

[1] Sokkary Nancy, Dietrich Jennifer E. (2017). Ovarian Embryology, Anatomy, and Physiology Including Normal Menstrual Physiology. Endocrine Surgery in Children.

[2] Allshouse Amanda A., Semple Amy L. (2016). Signs and Symptoms of Primary Ovarian Insufficiency. Primary Ovarian Insufficiency.

[3] Blackman JA. Midlife and beyond: female hormonal status/menopause and its effects on sexual health. In: Bartlik B, Espinosa G, Mindes J, editors. Integrative Sexual Health. Oxford: Oxford University Press; 2018. p. 28.

[4] Siegel RL, Miller KD, Jemal A (2018). Cancer statistics, 2018. CA Cancer J Clin.

[5] Luisi S, Orlandini C, Regini C, Pizzo A, Vellucci F, Petraglia F (2015). Premature ovarian insufficiency: from pathogenesis to clinical management. J Endocrinol Investig.

[6] Hoshiba T, Chen G, Endo C, Maruyama H, Wakui M, Nemoto E, Kawazoe N, Tanaka M (2016). Decellularized extracellular matrix as an in vitro model to study the comprehensive roles of the ECM in stem cell differentiation. Stem Cells Int.

[7] Scarritt ME, Pashos NC, Bunnell BA. A review of cellularization strategies for tissue engineering of whole organs. Front Bioeng Biotechnol. 2015;3:43. 10.3389/fbioe.2015.00043 PMC4378188 25870857

[8] Ji S, Guvendiren M. Recent advances in bioink design for 3D bioprinting of tissues and organs. Front Bioeng Biotechnol. 2017;5:23. 10.3389/fbioe.2017.00023 PMC5380738 28424770

[9] Kim Byoung Soo, Kim Hyeonji, Gao Ge, Jang Jinah, Cho Dong-Woo (2017). Decellularized extracellular matrix: a step towards the next generation source for bioink manufacturing. Biofabrication.

[10] Pati F, Cho DW (2017). Bioprinting of 3D tissue models using decellularized extracellular matrix bioink. Methods Mol Biol.

[11] Gibson Matt, Beachley Vince, Coburn Jeannine, Bandinelli Pierre Alain, Mao Hai-Quan, Elisseeff Jennifer (2014). Tissue Extracellular Matrix Nanoparticle Presentation in Electrospun Nanofibers. BioMed Research International.

[12] Rutz Alexandra L., Shah Ramille N. (2015). Protein-Based Hydrogels. Polymeric Hydrogels as Smart Biomaterials.

[13] Sawkins M, Bowen W, Dhadda P, Markides H, Sidney L, Taylor A, Rose F, Badylak S, Shakesheff K, White L (2013). Hydrogels derived from demineralized and decellularized bone extracellular matrix. Acta Biomater.

[14] Medberry CJ, Crapo PM, Siu BF, Carruthers CA, Wolf MT, Nagarkar SP, Agrawal V, Jones KE, Kelly J, Johnson SA (2013). Hydrogels derived from central nervous system extracellular matrix. Biomaterials.

[15] Wolf MT, Daly KA, Brennan-Pierce EP, Johnson SA, Carruthers CA, D'Amore A, Nagarkar SP, Velankar SS, Badylak SF (2012). A hydrogel derived from decellularized dermal extracellular matrix. Biomaterials.

[16] Hodgson MJ, Knutson CC, Momtahan N, Cook AD. Extracellular matrix from whole porcine heart decellularization for cardiac tissue engineering. In: Methods in Molecular Biology. Humana Press. 2017;31:1-8. 10.1007/7651_2017_31 28456953

[17] Porzionato A, Sfriso MM, Pontini A, Macchi V, Buompensiere MI, Petrelli L, Bassetto F, Vindigni V, De Caro R (2017). Development of small-diameter vascular grafts through Decellularization of human blood vessels. J Biomater Tissue Eng.

[18] Simões IN, Vale P, Soker S, Atala A, Keller D, Noiva R, Carvalho S, Peleteiro C, Cabral JM, Eberli D (2017). Acellular urethra bioscaffold: Decellularization of whole urethras for tissue engineering applications. Sci Rep.

[19] Consolo F, Brizzola S, Tremolada G, Grieco V, Riva F, Acocella F, Fiore GB, Soncini M (2016). A dynamic distention protocol for whole-organ bladder decellularization: histological and biomechanical characterization of the acellular matrix. J Tissue Eng Regen Med.

[20] Destefani AC, Sirtoli GM, Nogueira BV (2017). Advances in the knowledge about kidney Decellularization and repopulation. Front Bioeng Biotechnol.

[21] Fedecostante M, Onciu OG, Westphal KGC, Masereeuw R (2017). Towards a bioengineered kidney: recellularization strategies for decellularized native kidney scaffolds. Int J Artif Organs.

[22] Xu Y, Li D, Yin Z, He A, Lin M, Jiang G, Song X, Hu X, Liu Y, Wang J (2017). Tissue-engineered trachea regeneration using decellularized trachea matrix treated with laser micropore technique. Acta Biomater.

[23] Skolasinski S, Panoskaltsis-Mortari A. Decellularization of intact lung tissue through vasculature and airways using negative and positive pressure. In: Methods in Molecular Biology. Humana Press. 2017;32:1-9. 10.1007/7651_2017_32 PMC5895527 28656583

[24] Porzionato A, Sfriso MM, Pontini A, Macchi V, Petrelli L, Pavan PG, Natali AN, Bassetto F, Vindigni V, De Caro R (2015). Decellularized human skeletal muscle as biologic scaffold for reconstructive surgery. Int J Mol Sci.

[25] Chen Y, Geerts S, Jaramillo M, Uygun BE. Preparation of decellularized liver scaffolds and recellularized liver grafts. In: Methods in Molecular Biology. Humana Press. 2017;56:1–16 10.1007/7651_2017_56 PMC6688852 28735385

[26] Syed O, Walters NJ, Day RM, Kim HW, Knowles JC (2014). Evaluation of decellularization protocols for production of tubular small intestine submucosa scaffolds for use in oesophageal tissue engineering. Acta Biomater.

[27] Nowocin AK, Southgate A, Gabe SM, Ansari T (2016). Biocompatibility and potential of decellularized porcine small intestine to support cellular attachment and growth. J Tissue Eng Regen Med.

[28] Woodruff TK, Shea LD (2007). The role of the extracellular matrix in ovarian follicle development. Reprod Sci.

[29] Berkholtz Courtney, Shea Lonnie, Woodruff Teresa (2006). Extracellular Matrix Functions in Follicle Maturation. Seminars in Reproductive Medicine.

[30] Carrier, A., Investigating the Role of Extracellular Matrix Proteins in Ovarian Folliculogenesis and Ovarian Cancer. Electronic Thesis and Dissertation Repository. 3869. 2016. https://ir.lib.uwo.ca/etd/3869.

[31] Laronda MM, Jakus AE, Whelan KA, Wertheim JA, Shah RN, Woodruff TK (2015). Initiation of puberty in mice following decellularized ovary transplant. Biomaterials.

[32] Kawecki M, Labus W, Klama-Baryla A, Kitala D, Kraut M, Glik J, Misiuga M, Nowak M, Bielecki T, Kasperczyk A (2018). A review of decellurization methods caused by an urgent need for quality control of cell-free extracellular matrix’ scaffolds and their role in regenerative medicine. J Biomed Mater Res B Appl Biomater.

[33] Gilpin Anna, Yang Yong (2017). Decellularization Strategies for Regenerative Medicine: From Processing Techniques to Applications. BioMed Research International.

[34] Badylak SF (2014). Decellularized allogeneic and xenogeneic tissue as a bioscaffold for regenerative medicine: factors that influence the host response. Ann Biomed Eng.

[35] Kawasaki Takanori, Kirita Yuhei, Kami Daisuke, Kitani Tomoya, Ozaki Chisa, Itakura Yoko, Toyoda Masashi, Gojo Satoshi (2015). Novel detergent for whole organ tissue engineering. Journal of Biomedical Materials Research Part A.

[36] Ma Jinhui, Ju Zhihai, Yu Jie, Qiao Yeru, Hou Chenwei, Wang Chen, Hei Feilong (2018). Decellularized Rat Lung Scaffolds Using Sodium Lauryl Ether Sulfate for Tissue Engineering. ASAIO Journal.

[37] De Roo C, Lierman S, Tilleman K, Peynshaert K, Braeckmans K, Caanen M, Lambalk CB, Weyers S, T'Sjoen G, Cornelissen R. Ovarian tissue cryopreservation in female-to-male transgender people: insights into ovarian histology and physiology after prolonged androgen treatment. Reprod BioMed Online. 2017;​34(6):557–66. 10.1016/j.rbmo.2017.03.008 28372892

[38] Nguyen QT (2011). A source of human ovarian cortical tissue from sex assignment surgery. Rollins Undergraduate Res J.

[39] Van Den Broecke R, Van Der Elst J, Liu J, Hovatta O, Dhont M (2001). The female-to-male transsexual patient: a source of human ovarian cortical tissue for experimental use. Hum Reprod.

[40] Kashi AM, Tahemanesh K, Chaichian S, Joghataei MT, Moradi F, Tavangar SM, Najafabadi ASM, Lotfibakhshaiesh N, Beyranvand SP, Anvari-Yazdi AF (2014). How to prepare biological samples and live tissues for scanning electron microscopy (SEM). Galen Medical J.

[41] Campbell KL (1979). Ovarian granulosa cells isolated with EGTA and hypertonic sucrose: cellular integrity and function. Biol Reprod.

[42] Gupta SK, Mishra NC. Decellularization methods for scaffold fabrication. In: Methods in Molecular Biology. Humana Press. 2017;34:1–10. 10.1007/7651_2017_34 28550502

[43] Morris AH, Stamer DK, Kyriakides TR (2017). The host response to naturally-derived extracellular matrix biomaterials. Semin Immunol.

[44] Amorim CA, Shikanov A (2016). The artificial ovary: current status and future perspectives. Future Oncol.

[45] Shea LD, Woodruff TK, Shikanov A (2014). Bioengineering the ovarian follicle microenvironment. Annu Rev Biomed Eng.

[46] Amorim Christiani A. (2016). Artificial Ovary. Gonadal Tissue Cryopreservation in Fertility Preservation.

[47] El-Hayek S, Clarke HJ (2016). Control of oocyte growth and development by intercellular communication within the follicular niche. Results Probl Cell Differ.

[48] Li R, Albertini DF (2013). The road to maturation: somatic cell interaction and self-organization of the mammalian oocyte. Nat Rev Mol Cell Biol.

[49] Sánchez F, Smitz J (2012). Molecular control of oogenesis. Biochim Biophys Acta (BBA) - Mol Basis Dis.

[50] Ricciardelli C, Rodgers RJ (2006). Extracellular matrix of ovarian tumors. Semin Reprod Med.

[51] Watson LN, Mottershead DG, Dunning KR, Robker RL, Gilchrist RB, Russell DL (2012). Heparan sulfate proteoglycans regulate responses to oocyte paracrine signals in ovarian follicle morphogenesis. Endocrinology.

[52] Marei W, Ghafari F, Fouladi-Nashta A (2012). Role of hyaluronic acid in maturation and further early embryo development of bovine oocytes. Theriogenology.

[53] Joo S, Oh S-H, Sittadjody S, Opara EC, Jackson JD, Lee SJ, Yoo JJ, Atala A (2016). The effect of collagen hydrogel on 3D culture of ovarian follicles. Biomed Mater.

[54] Fu X-F, Cheng S-F, Wang L-Q, Yin S, De Felici M, Shen W (2015). DAZ family proteins, key players for germ cell development. Int J Biol Sci.

[55] Johnson J, Bagley J, Skaznik-Wikiel M, Lee H-J, Adams GB, Niikura Y, Tschudy KS, Tilly JC, Cortes ML, Forkert R (2005). Oocyte generation in adult mammalian ovaries by putative germ cells in bone marrow and peripheral blood. Cell.

[56] Bhartiya D, Shaikh A, Anand S, Patel H, Kapoor S, Sriraman K, Parte S, Unni S (2016). Endogenous, very small embryonic-like stem cells: critical review, therapeutic potential and a look ahead. Hum Reprod Update.

[57] Shaikh A, Anand S, Kapoor S, Ganguly R, Bhartiya D (2017). Mouse bone marrow VSELs exhibit differentiation into three embryonic germ lineages and germ & hematopoietic cells in culture. Stem Cell Rev Rep.

[58] Ratajczak MZ, Ratajczak J, Suszynska M, Miller DM, Kucia M, Shin DM (2017). A novel view of the adult stem cell compartment from the perspective of a quiescent population of very small embryonic-like stem cells. Circ Res.

[59] Guerin CL, Blandinieres A, Planquette B, Silvestre JS, Israel-Biet D, Sanchez O, Smadja DM (2017). Very small embryonic-like stem cells are mobilized in human peripheral blood during hypoxemic COPD exacerbations and pulmonary hypertension. Stem Cell Rev.

[60] Wojakowski W, Ratajczak MZ, Tendera M (2010). Mobilization of very small embryonic-like stem cells in acute coronary syndromes and stroke. Herz.

[61] Zbucka-Kretowska M, Eljaszewicz A, Lipinska D, Grubczak K, Rusak M, Mrugacz G, Dabrowska M, Ratajczak MZ, Moniuszko M (2016). Effective mobilization of very small embryonic-like stem cells and hematopoietic stem/progenitor cells but not endothelial progenitor cells by follicle-stimulating hormone therapy. Stem Cells Int.

[62] Olson DR, Blake CA (1991). Basal luteinizing hormone and follicle-stimulating hormone release rates as a function of time after castration in female and male rats. Neuroendocrinology.

[63] Eldridge JC, Dmowski WP, Mahesh VB (1974). Effects of castration of immature rats on serum FSH and LH, and of various steroid treatments after castration. Biol Reprod.

[64] Alexandris E, Milingos S, Kollios G, Seferiadis K, Lolis D, Messinis IE (1997). Changes in gonadotrophin response to gonadotrophin releasing hormone in normal women following bilateral ovariectomy. Clin Endocrinol.

[65] Wise PM, Ratner A (1980). Effect of ovariectomy on plasma LH, FSH, estradiol, and progesterone and medial basal hypothalamic LHRH concentrations old and young rats. Neuroendocrinology.

