# Supplementary figures and images for: Decellularized human ovarian scaffold based on a sodium lauryl ester sulfate (SLES)-treated protocol, as a natural three-dimensional scaffold for construction of bioengineered ovaries
Source: Stem Cell Res Ther. 2018 Sep 26;9:252. doi: 10.1186/s13287-018-0971-5 (PMC6158855; doi:10.1186/s13287-018-0971-5)

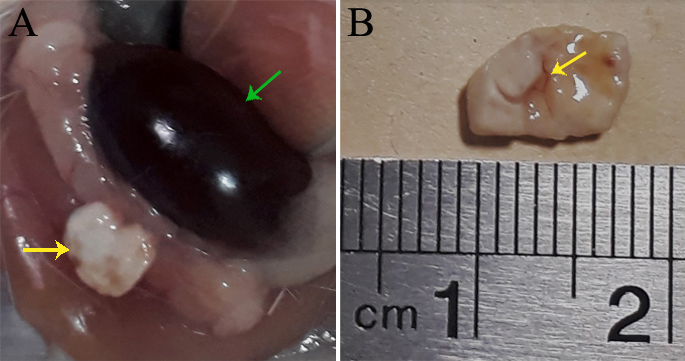

Supplement: Supplementary file 1 — Macroscopic observation of the transplanted grafts. (A): The grafts (yellow arrow) were sutured onto renal (green arrow) fat pad of ovariectomized immature rats bilaterally. (B): Gross observation showed a vascular structure (yellow arrow) and there was no sign of rejection 4 weeks after the surgery. (TIF 1470 kb) [file 13287_2018_971_MOESM1_ESM.tif]

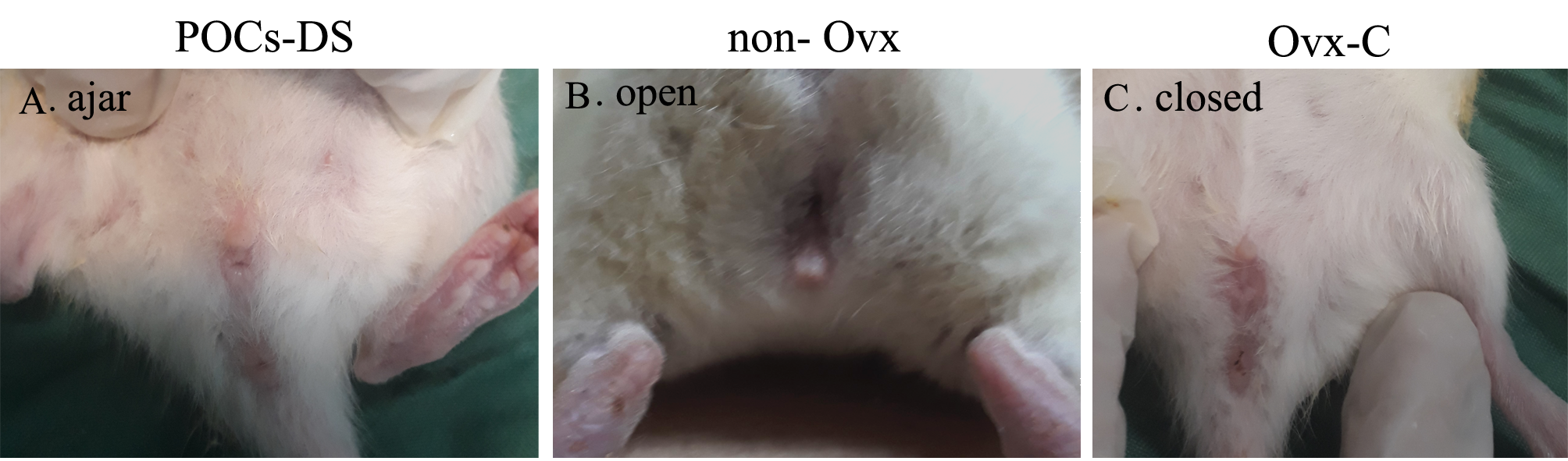

Supplement: Supplementary file 2 — Vaginal patency as a criterion of pubertal transition. The vaginal orifice of OVX-C animals was imperforated with a visible hymen (C), while the orifice of the rats receiving POCs-DS grafts (A) was found to be open, but less than that of the non-OVX animals (B). OVX-C ovariectomized control, non-OVX non-ovariectomized, POCs-DS primary ovarian cells seeded decellularized scaffolds. (TIF 6975 kb) [file 13287_2018_971_MOESM2_ESM.tif]
